# Economic costs of health and social care for a child with a life-limiting condition in their last year of life: a systematic review

**DOI:** 10.1136/bmjpo-2025-003526

**Published:** 2025-07-16

**Authors:** Louise Prendergast, Ellie Crane, Nathan Bray, Jane Noyes

**Affiliations:** 1 School of Health Sciences, Bangor University, Bangor, Gwynedd, UK

**Keywords:** Palliative Care, Infant, Health services research

## Abstract

**Aim:**

To systematically review and descriptively synthesise the costs associated with any health and/or social care services provided to children with a life-limiting condition, in their last year of life.

**Methods:**

Systematic review using MEDLINE, EMBASE, CENTRAL Cochrane Library, CINAHL and other specialist databases to identify articles published between 2004 and 2024. Duplicates were removed and two authors screened the articles and abstracts independently. Full-text articles were assessed for eligibility, with quality assessment conducted using the Drummond checklist. The findings were exported into tables and summarised narratively. Costs from each study were calculated per person per month and inflated to 2024 US dollars.

**Results:**

20 records were eligible for final inclusion. Services reported were varied as were approaches to estimating costs. In general, younger children required more intensive and higher cost care. Costs increase in the final months of life, and specialist paediatric palliative care interventions were associated with overall lower costs through reductions in hospital admissions and length of stay.

**Conclusions:**

It was not possible to present a reliable robust estimate of the economic costs of services and resources due to limited and heterogenic data. Further research is needed to identify the full range of resources, and services associated with caring for children with life-limiting conditions in their last year of life, to support healthcare planning and resource allocation.

**WHAT IS ALREADY KNOWN ON THIS TOPIC**

- There is a lack of systemic research that documents the economic costs associated with supporting children with a life-threatening or life-shortening condition in their last year of life.
- Health and social care systems are under pressure to deliver care and support that provides value for money and to use existing resources efficiently.
- To effectively plan paediatric palliative care services and forecast future expenses, it is essential to identify the current resources and costs.

**WHAT THIS STUDY ADDS**

- This review shows that there is limited research exploring the economic costs of care for children with a life-limiting condition.
- The range of resources identified and costed in the studies is highly varied, as are the study designs.
- Further research is needed to comprehensively identify all resources associated with the last year of life of a child with a life-limiting condition, the associated costs and the bearer of these.

## Background

 The number of children with life-limiting conditions has increased over the last 20 years in the UK and other high-income countries. The care and support needs for these children are evolving due to advances in public health, medical and nursing care and technology.^1 2^

Paediatric palliative care differs from adult palliative care as it is usually delivered over a longer time frame and associated with different resource utilisation.^2^,^4^ Around 15% of children receiving palliative care have no underlying diagnosis, and there is no ‘typical pathway’ to describe their care needs.^5 6^ Paediatric palliative care may start at diagnosis and last for days, months or years, continuing regardless of whether the child is actively receiving curative treatment.^3 7 8^ The latter model of combining palliative care with curative treatment is called parallel planning in the UK, similar to concurrent hospice care in the USA. Variations exist in the provision of services, professionals involved and funding sources across different settings, including the home, hospital and hospice.^5^ Resource use is often high, expensive and unpredictable due to the need for multiple tailored care plans and a mix of services to suit the health and social care needs of each child. This can make estimating costs a challenge. Understanding the resources, services and their associated economic costs is essential to underpin effective planning to provide quality and cost-effective care. Therefore, to explore these costs, we conducted a systematic review to identify and synthesise international evidence on the economic costs associated with health and social care for children with a life-limiting condition in the last year of life.

## Methods

### Study design

A systematic review was conducted in accordance with the Cochrane Handbook for Systematic Reviews of Interventions (V.6.5, 2024), including the guidance for incorporating economic evidence in chapter 20.^9 10^ Reporting followed relevant domains of the Preferred Reporting Items for Systematic Reviews and Meta-Analyses reporting checklist.^11^

The review question was ‘What are the economic costs associated with care for children with a life-limiting condition in their last year of life?’ A review protocol was developed (online supplemental file 1); however, this protocol was not registered.

## Identifying relevant studies

### Search strategy

Search terms and search strategy were identified and refined by the authors (online supplemental file 2), and the search strategy was structured using the SPICE (setting, perspective, intervention/phenomenon of interest, comparator and evaluation) framework (table 1).^12^ The search strategy used in MEDLINE was Palliative Care OR Terminally Ill/ Terminal Care OR Hospice Care OR life limiting condition OR life limiting illness AND Pediatric/paediatric OR Child OR Adolescent OR Infant/Newborn AND Health Care Economics and Organizations OR Models, Economic OR Cost-Benefit Analysis OR Health Care Costs OR Delivery of Health Care OR Cost Savings OR Costs and cost analysis OR budgets. These same search terms were used and adapted to database-specific subheadings in EMBASE, Cochrane Library and CINAHL (see online supplemental file 2). The following specialist repositories for paediatric palliative care were also searched: Together for Short Lives (www.togetherforshortlives.org.uk) and the Paediatric Palliative Care Library (https://pedpalascnetlibrary.omeka.net/). A search of the first five pages was also conducted in Google Scholar, and reference lists of relevant articles were screened.

**Table 1:** SPICE framework

Setting	High-income countries with a healthcare system comparable to the UK National Health Service (NHS). The setting includes hospitals, hospices or community-based care within the home.
Perspectives	Health and social care service providers.
Phenomenon of interest	The costs of care and resources provided to a child with a life-limiting condition in their last year of life from any core, generalist and/or specialist service.
Comparator	Comparing the costs of care in the last 12 months of life between intervention and comparator groups, as well as cost differences of care based on demographic, geographic and setting variables such as location of care, age of the child or condition types.
Evaluation	Systematic review to identify studies, aggregate and describe the evidence.

SPICE, setting, perspective, intervention/phenomenon of interest, comparator and evaluation.

## Inclusion and exclusion criteria

Papers were screened according to the inclusion and exclusion criteria presented in table 2.

**Table 2:** Inclusion and exclusion criteria

Inclusion	Exclusion
Studies including paediatric patients up to 21 years of age with any life-limiting condition.	Studies that focused only on children with non-life-limiting conditions.
Studies that reported on the economic costs associated with health and/or social care services and/or resources provided to children within their last year of life.	Studies that reported exclusively on neonates (a child under 28 days). This was due to the distinct care pathways and interventions associated with neonatal children.
Studies in high-income countries with healthcare systems comparable to the United Kingdom.	Studies that reported in languages other than English or Welsh
Studies published between January 2004 and November 2024.	Studies that reported exclusively on the costs associated with support from the family.
Studies conducted within any health/social care setting, including but not limited to hospital, home or hospice settings.	Studies in the form of editorials, letters or commentaries.
Studies reported in English or Welsh.	

## Study selection

Search results were imported into Mendeley where duplicates were automatically removed. Titles and abstracts were first screened against the eligibility criteria by one author (LP). The full texts of potentially relevant papers were then assessed independently by two authors (LP and EC). Disagreements were resolved through discussion with a third reviewer (NB or JN).

## Critical appraisal

The Drummond checklist, designed for appraising economic evaluations, was used to guide the critical assessment of the studies.^13^ Two authors (LP and EC) independently assessed the studies according to the ten statements.

## Data extraction

Data were extracted from the included studies by the authors (LP and EC) using a dual data extraction process. The following variables were extracted from included studies.

- Author, year of publication and country.
- Study aims.
- Study methods.
- Characteristics of children included in the study.
- Resources included in the costing estimate.
- Data sources used to estimate resource utilisation and costs.
- Cost of health and social care associated with caring for a child in the last year of life.

### Data analysis and synthesis

The health and social care costs associated with caring for a child in their final year of life were extracted from each included study. Where necessary, total costs were converted to a per-person-per-month (PPPM) figure to enable comparison across studies. All costs were standardised to 2024 dollars (USD$). Published costs were first inflated to 2024 values using the country-level Consumer Price Index (onlinesupplemental file 2 table S1). If the study cost year was reported, this was used as the baseline year for the adjustment to 2024 costs. If the cost year was not reported, we used the final year of data collection as a baseline to adjust prices to 2024. After adjusting for inflation, if costs were reported in a different currency, we converted them to USD using the exchange rates as of November 2024 (online supplemental file 2, tables S2 and S3).

Ideally, we wanted to pool costing estimates. Due to heterogeneity in methodology and reporting across the included studies, economic meta-analysis was not possible. Therefore, we analysed the economic evidence on the costs associated with health and social care in the final year of a child’s life descriptively. Costing data were summarised narratively to identify patterns and trends across studies. Where possible, costing data were grouped by variables such as life-limiting condition, age of the child, care settings and country.

### Patient and public involvement statement

Patients and public were not involved in this review.

## Results

### Search results

**Figure 1:**
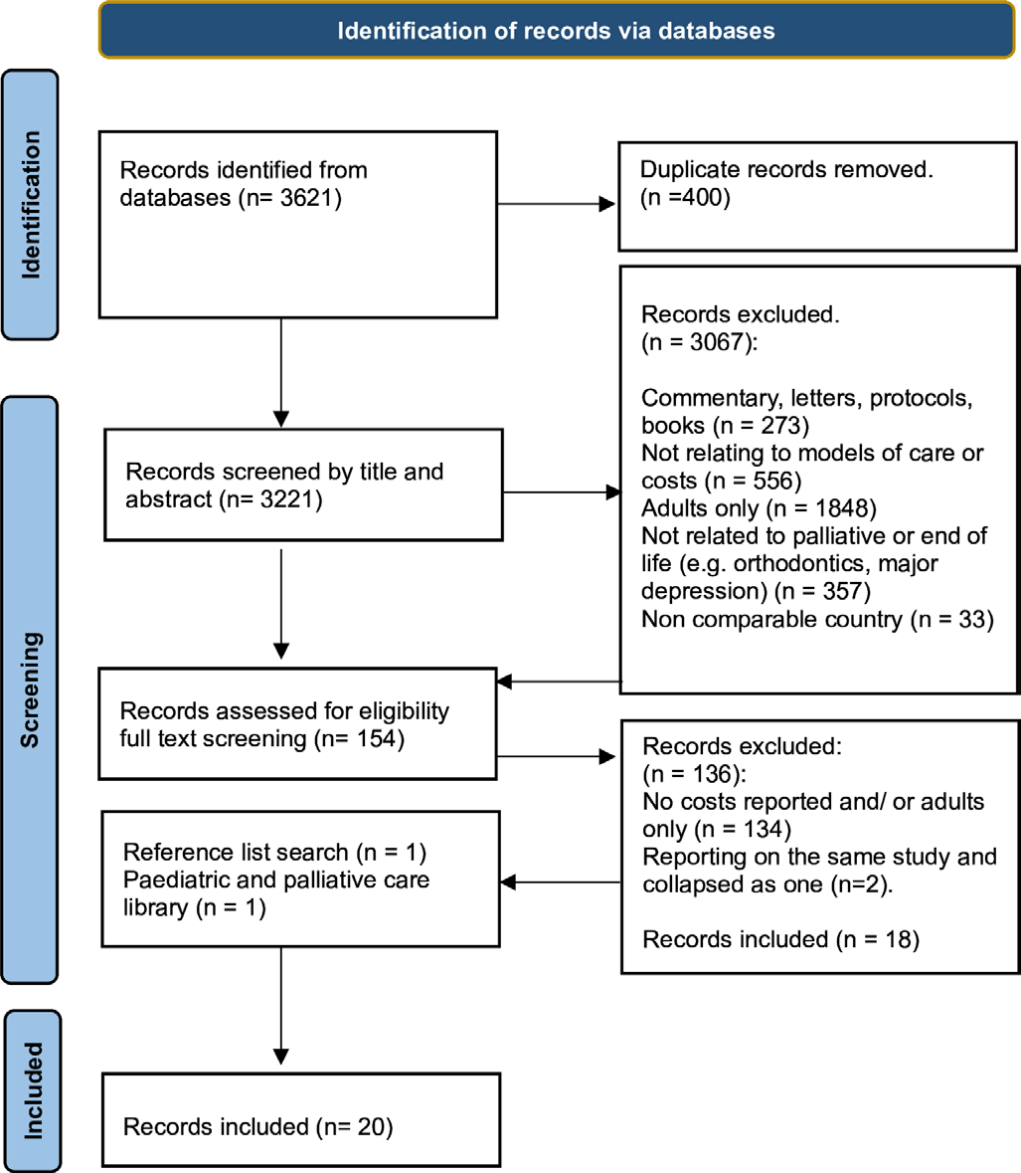
Flowchart of study selection.

A total of 3621 articles were identified. Following the removal of 400 duplicates, 3221 articles remained. After title and abstract screening, 154 papers were retrieved and deemed eligible for full-text screening, after which a further 136 articles were excluded (figure 1). Because two articles reported on the same study, we treated them as a single reference.^14 15^ Two articles were identified outside of the database searches. Therefore, a total of 20 studies were included (figure 1).

## Characteristics of included studies

Most studies were conducted in the USA (n=12), followed by Canada (n=5), the United Kingdom (n=2) and Singapore (n=1) (table 3). The cohort size of paediatric patients ranged from less than 10 children to 18 152 children. Ages ranged from newborn to 21 years of age. Six studies included children with complex chronic conditions (CCCs) using the ICD-9-CM organ-based system^16^,^20^ or ICD-10 organ-based system.^21 22^ Two studies described children with CCCs without applying a specific framework.^23 24^ Four studies focused on children with cancer.^25^,^28^ Two studies defined conditions by the Together for Short Lives/Royal College of Paediatrics and Child Health categories of life-limiting conditions.^29 30^ One study investigated spinal muscular atrophy type 1.^31^ Four studies examined costs without specifying condition criteria or characteristics.^1532^,^34^

**Table 3:** Characteristics of included studies

Study and country	Study methods and number of children	Conditions of children included	Age of children in the study
Ananth *et al*USA^20^	Cohort1250	Life-threatening complex chronic conditions (LT-CCCs) identified with (ICD-9CM)	Children aged 1–18 years; median age=8 years
Chirico *et al*USA^23^	Cohort224	Complex chronic conditions	Aged 0–21 years; mean age at death=7.4 years
Chong *et al*Singapore^29^	Cohort138 (control group=67; treatment group=71)	Life-limiting condition defined by Together for Short Lives criteria. Neonates excluded	Less than 19 years of age at time of diagnosis. Mean age at death was 12.2 for the treatment group and 6.3 for the control group
Cozad *et al*USA^24^	Cohort18 147	Complex chronic conditions	Children under 21 years; average age=7 years
de Oliveira *et al*Canada^25^	Cohort6725	Children and adolescents diagnosed with cancer	Children aged 91 days to 15 years, and adolescents aged 15–19 years. Mean ages at diagnosis were 6 years for children, and 17 years for adolescents
de Oliveira *et al*Canada^26^	Cohort7049	Children and adolescents diagnosed with cancer	Children aged 0 days to 14.9 years, and adolescents aged 15–19.9 years. Mean ages at diagnosis were 6 years for children, and 17 years for adolescents
Gans *et al*USA^15^	Programme evaluation132	Children with life-limiting illness	Children aged <20 years
Knapp *et al*USA^22^	Observational1431	Categorised into four condition categories based on the International Classification of Disease, Tenth Revision (ICD-10)	Average age for infants (<1 year) 4.4 months and for children (1–21 years) was 11.2 years
Lemoine et alUSA^31^	Cohort49	Children with spinal muscular atrophy type 1	Mean age at death was 7.6 years in the proactive care group, and 8.8 in the supportive care group
Lindley *et al*USA^16^	Cohort1423	Complex chronic conditions categorised by the ICD9-CM organ-based conditions	Children aged 0–20 years
Lindley *et al*USA^17^	Cohort17 062	Complex chronic conditions categorised by the ICD9-CM organ-based conditions	Children aged between 0 and 17
Lindley *et al*USA^18^	Cost-effectiveness analysis18 152	Complex chronic conditions categorised by the ICD9-CM organ-based conditions	Children under 21 years
Lysecki *et al*Canada^34^	Cohort3967	Children who died in Ontario Canada	Children <19 years at the time of death
Nathan *et al*Canada^27^	Cohort1356	Diagnosed with cancer	Children aged 15–17 at diagnosis. Mean age treated in adult institution 16.6 years, paediatric institution 15.7 years
Noyes *et al*UK^30^	Multi-method epidemiological and economic exemplar1052	Children with complex health and palliative care needs defined by Together for Short Lives/RCPCH categories	Under the age of 19
McFerran *et al*UK^28^	Cost consequence analysis< 10	Diagnosed with cancer	Children aged 0–19 years
Smith *et al*USA^19^	Cohort902	Classified using the ICD9-CM organ system categories for complex chronic conditions	Not specified
Svynarenko *et al*USA^32^	Cost-effectiveness analysis18 152	Children with a prognosis of 6 months or less to live	Children under 20 years
Svynarenko *et al*USA^33^	Incremental cost analysis1788	Paediatric patients who had died	Children under 20 years; mean age=9 years
Widger *et al*Canada^21^	Cohort1620	Children who died in Ontario categorised into four condition categories based on the ICD-10 classification system	Children aged 30 days to 19 years

ICD9-CM, International Classification of Diseases, 9th Revision, Clinical Modification; RCPCH, Royal College of Paediatrics and Child Health; SMA, spinal muscular atrophy.

### Quality of studies

Quality assessments were conducted using the Drummond checklist, and all studies were rated as being average or good in quality (online supplemental file 3).

### Costing information and methods

There was wide variation in the resources included in the costing analyses and data sources used. Ten studies estimated costs in the last year of life using data from healthcare insurance claims, with eight of these relying specifically on Medicaid insurance data (table 4).^15^,^1822^ Among these, insurance claims were generally assumed to provide a comprehensive estimate of care costs in the last year of life, however, these lacked details about the resources included in the final costing estimate. For example, reported Medicaid care costs in the USA differed between studies, with reasons for these differences unclear. Studies that did not rely solely on insurance claims typically used a combination of administrative databases and cost unit information to estimate costs across a broad range of care variables.^2021 25^,^28 30 34^ Other studies utilised financial statements for their cost analyses.^19 29^

**Table 4:** Costs of paediatric end-of-life care (per patient per month, USD 2024): concurrent versus standard hospice care

Study	Resources included in the costing estimate	Data sources used to estimate costs	Costs (per patient per month, reported in 2024 US dollars)
Cozad *et al*^24^	Inpatient care, outpatient care and prescription drugs.	Medicaid administrative data expenditures	Concurrent hospice care group: $8537 Standard hospice care group: $6090
Lindley *et al*^18^	Person-level data for itemised costs of care (hospitalisations and hospice care).	Medicaid data.	Concurrent hospice care: $6090 Standard hospice care: $8537
Svynarenko *et al*^32^	Costs in last year of life for concurrent hospice care or standard hospice care. Specific costs included were not specified.	Medicaid costs	Urban location: Concurrent hospice care: $2590 PPPM (95% CI: $1818 to $3362) Standard hospice care: $1503 PPPM (95% CI: $1313 to $1694) Rural location: Concurrent hospice care: $5298 PPPM (95% CI: $4321 to $6277) Standard hospice care: $3871 PPPM (95% CI: $3536 to $4206)
Svynarenko *et al*^33^	Costs in last year of life for concurrent hospice care or standard hospice care. Specific costs included were not specified.	Medicaid costs	Standard care: Aged under 1: $3921 (95% CI: $3602 to $4533) Aged 1–5 years: $6145 (95% CI: $4891 to $7619) Aged 6–14 years: $7169 (95% CI: $5304 to $7631) Aged 15–20 years: $6630 (95% CI: $5237 to $8454) Concurrent care: Aged under 1: $5907 (95% CI: $5077 to $6827) Aged 1–5 years: $7198 (95% CI: $5912 to $8241) Aged 6–14 years: $11 481 (95% CI: $8425 to $11 838) Aged 15–20 years: $9037 (95% CI: $8128 to $11 694)

PPPM, per patient, per month.;

Some studies estimated the costs of one specific aspect of care, such as unscheduled emergency hospital care or inpatient costs.^19 28^ However, most studies included a broader and more comprehensive estimation of all care costs provided during this time, including both the costs of active treatment and palliative care. These estimates typically included hospice care, home care, outpatient visits, inpatient costs, emergency department (ED) visits, therapies, diagnostic tests and medications.

## Cost

There was a broad range in the cost of care for a child in the last year of life across the included studies (tables4^7^). As noted in the methods section, all costs are reported in USD$ (standardised to 2024) unless otherwise stated. Costs ranged from an estimate of $1290 PPPM for unscheduled hospital emergency care for children with cancer in the UK^27^ to $61 200 PPPM for all hospital resource use and major medical interventions for children with haematologic or immunologic conditions in the USA.^20^ This variation likely reflects differences in the study methodologies, healthcare systems, patient populations and models of care.

**Table 5:** Costs of paediatric end-of-life care (per patient per month, USD 2024) by patient age

Study	Resources included in the costing estimate	Data sources used to estimate costs	Costs (per patient per month, reported in US dollars, 2024)
de Oliveira *et al*^25^	Inpatient hospitalisations, emergency department, same-day surgeries, outpatient chemotherapy, radiation therapy, outpatient diagnostic and laboratory tests, physician services, home care services, outpatient prescription drugs.	Costs from the perspective of the public payer (Ontario Ministry of Health and Long-Term Care). Combination of databases, including, the Canadian Institute for Health Information (CIHI), OHIP Claims History, Cancer Care Ontario, and the Ontario Drug Benefit Program.	Adolescents (aged 15–19.9 years): All cancer types: $16 747 (95% CI: $13 410 to $20 081) Bone and soft tissue cancer: $12 849 (95% CI: $9772 to $15 926) Germ cell cancer: $9458 (95% CI: $4345 to $14 548) Lymphoma: $20 616 (95% CI: $14 071 to $27 162) Central nervous system cancer: $13 982 (95% CI: $8338 to $19 625) Leukaemia: $25 191 (95% CI: $14 637 to $35 745) All other cancers: $7193 (95% CI: $3756 to $10 630) Children (91 days to 14 years): All cancer types: $19 105 (95% CI: $16 993 to $21 297) Lymphoma: $29 544 (95% CI: $16 960 to $42 128) Leukaemia: $26 352 (95% CI: $21 022 to $31 681) All other cancers: $19 776 (95% CI: $16 501 to $23 052)
Lindley *et al*^16^	Hospital inpatient, prescription drugs, hospice/home health, primary care, other acute care, ancillary care.	Medicaid data	Overall: $16 345 Aged under 1: $23 758 Aged 1–5 years: $20 588 Aged 6–14 years: $12 823 Aged 15–20 years: $12 728
Lindley *et al*^17^	Hospice use, hospice length of stay, primary care visit, hospital readmissions and emergency department.	Insurance claims	Aged<1 year: $14 782, Aged 1–5 years: $3837 Aged 6–14 years: $3698 Aged 15–17 years: $3432 (Expenditures only)
Knapp *et al*^22^	Costs for inpatient, outpatient and hospice services. Hospice expenditures included both home and inpatient hospice care.	Medicaid claims	Children (over 1 year of age): $8059 Infants (under 1 year of age): $12 999
Svynarenko *et al*^33^	Costs in last year of life for concurrent hospice care or standard hospice care. Specific costs included were not specified.	Medicaid costs	Standard care: Aged under 1: $3921 (95% CI: $3602 - $4533) Aged 1–5 years: $6145 (95% CI: $4891 to $7619) Aged 6–14 years: $7169 (95% CI: $5304 to $7631) Aged 15–20 years: $6630 (95% CI: $5237 to $8454) Concurrent care: Aged under 1: $5907 (95% CI: $5077 to $6827) Aged 1–5 years: $7198 (95% CI: $5912 to $8241) Aged 6–14 years: $11 481 (95% CI: $8425 to $11 838) Aged 15–20 years: $9037 (95% CI: $8128 to $11 694)

**Table 6:** Costs of paediatric end-of-life care (per patient per month, USD 2024): specialised care

Study	Resources included in the costing estimate	Data sources used to estimate costs	Costs (per patient per month, reported in US dollars, 2024)
Chirico *et al*^23^	Paediatric palliative care programme which includes social work case managers, paediatric nurses, practitioners/registered nurses, child life specialists and palliative care physicians.	Costing data obtained from insurance claims warehouse of the Lifetime HealthCare Companies	Death at home: $13 323 (95% CI: $10 346 to $17 155) Death at the hospital: $22 004 (95% CI: $17 796 to $27 210)
Chong *et al*^29^	Costs-per-patient at the end of life including both healthcare and intervention costs. Healthcare costs referred to the cost of healthcare resources used, including hospitalisations, emergency department visits and outpatient visits. For the treatment group, the costs of the paediatric palliative care hospice intervention were also added.	For the treatment group, costs of intervention were obtained from Star PALS’s financial statements.	Control group (treatment as usual: $18 038 Intervention group (home-based palliative care): $5321
Gans *et al*^15^	Inpatient, outpatient, emergency department and pharmacy.	Medicaid claims and programme enrolment data costs	Control group (treatment as usual): $20 962 Intervention group (home-based family-centred care): $16 498
Lemoine *et al*^31^	Inpatient, outpatient and emergency department care at a tertiary care hospital and costs from across twenty other hospitals or outpatient clinics.	Medicaid claims	Whole study sample: $6290 (95% CI: $1371 to $10 827) Control group: $4900 (95% CI: $1102 to $11 103) Intervention group (proactive care): $7469 (95% CI: $4819 to $10 827)
Lysecki *et al*^34^	Acute and home care, including, costs for emergency department, same day surgery, inpatient settings, neonatal and paediatric intensive care units (ICU), non-ICU wards, mental health units, alternate level of care and home and outpatient care.	Administrative data sources to estimate costs, including the Ontario Registered Persons Database, Canadian Institute for Health Information databases, Ontario Mental Health Reporting System, Continuing Care Reporting System, National Rehabilitation System, Ontario Health Insurance Plan Claims Database, Ontario Drug Benefit Claims Database and Home Care Database.	Children in region with no specialist paediatric palliative care (SPPC-) services: $6698 Children in region with specialist paediatric palliative care (SPPC+) services: $5246 Rural location: $2009 (IQR: $149 to $11 526) Urban location: $576 (IQR: $93 to $5473)
Noyes *et al*^30^	Costs of palliative care for children, including specialist resources such as palliative care nurses, medical equipment technician, clinical psychologist, IT support, administrative support, 24/7 telephone nurse consultation and NHS-provided services.	Unit Costs of Health and Social Care 2009, NHS reference costs for 2006–2007 and for 2007–2008, and the Agenda for Change pay scale.	$3975 $2662 additional week at home
Smith *et al*^19^	Inpatient costs.	Cost accounting system of Intermountain Healthcare	10% most costly paediatric palliative care patients: $16 350

NHS, National Health Service.

**Table 7:** Costs of paediatric end-of-life care (per patient per month, USD 2024): conditions

Study	Resources included in costing estimate	Data sources used to estimate costs	Costs (per patient per month, reported in US dollars, 2024)
Ananth *et al*^20^	Hospital resource use and major medical interventions. Costs calculated from number of hospital admissions, aggregate days in the hospital and hospital costs for medical interventions including mechanical ventilation, surgery and new use of medical technology in the terminal admission.	Paediatric Health Information System (PHIS) database.	Overall costs for all patients: $16 157 (IQR: $5131 to $46 477) Cardiovascular conditions: $19 380 (IQR: $5780 to $53 607) Congenital/genetic conditions: $16 433 (IQR: $6460 to $36 493) Gastrointestinal conditions: $54 740 (IQR: $22 667 to $84 773) Haematologic/immunologic conditions: $61 200 (IQR: $28 333 to $99 620) Malignancy conditions: $31 507 (IQR: $12 013 to $65 960) Metabolic conditions: $42 840 (IQR: $16 433 to $82 960) Neuromuscular conditions: $12 353 (IQR: $4307 to $32 867) Renal conditions: $41 707 (IQR: $16 660 to $92 593) Respiratory conditions: $33 886 (IQR: $14 053 to $66 640)
Chirico *et al*^23^	Paediatric palliative care programme which includes social work case managers, paediatric nurses, practitioners/registered nurses, child life specialists and palliative care physicians.	Costing data from insurance claims warehouse of the Lifetime HealthCare Companies	Malignancy conditions: $21 401 (95% CI: $16 600 to $27 592) All other conditions: $13 699 (95% CI: $11 092 to $16 917)
de Oliveira *et al*^25^	Inpatient hospitalisations, emergency department, same-day surgeries, outpatient chemotherapy, radiation therapy, outpatient diagnostic and laboratory tests, physician services, home care services, outpatient prescription drugs.	Combination of databases, including, the Canadian Institute for Health Information (CIHI), OHIP Claims History, Cancer Care Ontario and the Ontario Drug Benefit Program.	Adolescents (aged 15–19.9 years): All cancer types: $16 747 (95% CI: $13 410 to $20 081) Bone and soft tissue cancer: $12 849 (95% CI: $9772 to $15 926) Germ cell cancer: $9458 (95% CI: $4345 to $14 548) Lymphoma: $20 616 (95% CI: $14 071 to $27 162) Central nervous system cancer: $13 982 (95% CI: $8338 to $19 625) Leukaemia: $25 191 (95% CI: $14 637 to $35 745) All other cancers: $7193 (95% CI: $3756 to $10 630) Children (91 days to 14 years): All cancer types: $19 105 (95% CI: $16 993 to $21 297) Lymphoma: $29 544 (95% CI: $16 960 to $42 128) Leukaemia: $26 352 (95% CI: $21 022 to $31 681) All other cancers: $19 776 (95% CI: $16 501 to $23 052)
de Oliveira *et al*^26^	Inpatient hospitalisations, emergency department visits, outpatient visits, chemotherapy visits and same day surgery.	OHIP Claims database record, shadow-billed records, the Ontario Drug Benefit Program, and the New Drug Funding Program data.	All cancer types: $35 127 (95% CI: $31 345 to $38 908) Central nervous system cancer: $31 195 (95% CI: $23 310 to $39 080) Leukaemia: $48 036 (95% CI: $40 322 to $55 751) Lymphoma: $41 432 (95% CI: $31 536 to $51 328) All other cancers: $27 831 (95% CI: $22 747 to $32 915)
McFerran *et al*^28^	Unscheduled emergency care costs.	Northern Ireland's General Registrar’s Office linked by cancer diagnosis to the Patient Administration System to identify admissions. Data mining to cost palliative care support and GP/ambulance involvement per admission.	$1290
Nathan *et al*^27^	Hospitalisations, emergency department visits, same-day surgeries, outpatient chemotherapy, radiation, diagnostic/laboratory tests, physician services, home care.	Combination including Ontario Health Insurance Plan Claims Database, National Ambulatory Care Reporting System, Discharge Abstract Database, Cancer Care Ontario Activity Level Reporting System.	Bone and soft tissue cancer: $22 438 (IQR: $9019 to $43 054) Germ cell cancer: $26 619 (IQR: $11 129 to $55 830) Lymphoma: $22 570 (IQR: $9603 to $60 458) Central nervous system cancer: $14 661 (IQR: $8491 to $35 937) Leukaemia: $54 505 (IQR: $17 801 to $149 541) All other cancers: $15 699 (IQR: $9563 to $28 086)
Widger *et al*^21^	Homecare, rehabilitation, acute care, inpatient admission, intensive care unit admissions, emergency department, outpatient clinic, physician and non-physician services.	Records of healthcare use paid for by the provincial Ministry of Health and Long-Term Care.	Perinatal/congenital cause of death: $13 122 Chronic illness: $8805 External cause of death: $1022 Other causes of death: $1559

### Comparing costs between concurrent versus standard hospice care

Four studies explored cost differences between concurrent and standard hospice care in the USA, using Medicaid insurance claims to estimate costs^18 24 32 33^ (table 4). Concurrent hospice care allows patients to access hospice care alongside standard medical care and typically includes active treatment as well as palliative care for symptom management and holistic support.

Both Lindley *et al* and Cozad *et al*^24^ analysed the same data set and reported that concurrent care was associated with higher Medicaid expenditures compared with standard hospice care. However, cost savings were demonstrated for children enrolled in concurrent hospice care for 1–14 days with reductions in total and inpatient care costs.^24^

Svynarenko *et al*^24^ examined urban and rural settings and found that concurrent hospice care was more expensive in both settings compared with standard hospice care. In rural regions, standard hospice care costs a mean of $3871 PPPM compared with $5298 PPPM for concurrent, and in urban regions, standard care costs a mean of $1503 PPPM compared with $2590 PPPM for concurrent care. Similarly, Svynarenko^33^ found that concurrent hospice care incurred higher costs than standard hospice care but was associated with a reduction in live hospice discharge across all age groups. The cost difference between standard and concurrent hospice care was highest for children aged 6–14 years, with the former costing $7169 PPPM and latter costing $11 481 PPPM, a $4312 PPPM difference. The authors concluded that this age group likely experiences higher rates of CCCs leading to increased treatment costs.

## Patient age and cost observations

Five studies analysed costs across different age groups (table 5).^16 22 25 33^ In general, infants and very young children incurred higher costs compared with older children and adolescents. This trend was attributed to younger children typically having a higher prevalence of complex or severe conditions, higher frequency of hospitalisations and increased need for intensive interventions.^16 17 33^

Knapp *et al*^22^ conducted an economic analysis of healthcare expenditure patterns for children during their last year of life, while Lindley *et al*^16^ explored the demographic characteristics, healthcare utilisation and expenditures for children in their last year of life. Both studies found that children under 1 year of age had substantially higher costs compared with older children. Lindley *et al* reported costs of $23 758 PPPM for infants under 1 year compared with $12 728 PPPM for children aged 15–20 years. Similarly, de Oliveira *et al*^25^ conducted a cohort study in Canada and found that, across all cancer types, children aged 91 days to 14 years incurred higher costs than adolescents aged 15–19 years. de Oliveira *et al*^25^ attributed the cost differences between children and adolescents to the intensity and type of treatments required during end-of-life care, with costs for children predominantly driven by inpatient hospitalisation, physician services and chemotherapy. Lindley *et al*^17^ in their retrospective cohort study found that infants under 1 year had higher hospice stays with a high frequency of hospital readmission and were the costliest of all the age groups.

This trend was not observed in an incremental cost analysis of standard hospice care compared with concurrent hospice care by Svynarenko *et al*.^33^ . In their study, younger age groups (under 1 and 1–5 years) had lower monthly costs compared with older children (6–14 years and 15–19 years). The authors hypothesised that this may be due to the early detection and management of congenital anomalies in younger children, potentially addressed more cost-effectively than complex conditions more common in older children. However, it is not fully clear why these findings diverge from other studies where younger children incurred higher costs.

### Costs of specialised paediatric palliative care

Seven studies investigated the costs of specialised services for children with life-limiting conditions.^15 19 23 29 31 34^ Proactive or community-based interventions emerged as a common specialised intervention (table 6)

Lemoine *et al* assessed costs for children with spinal muscular atrophy type 1 and found that initiating early non-invasive respiratory care was linked to longer survival times, which subsequently increased overall healthcare costs due to extended care periods.

Smith *et al*^19^ examined the 10% most costly paediatric inpatients and discovered that paediatric palliative care did not significantly change inpatient costs compared with no paediatric palliative care. The authors suggest that paediatric palliative care can enhance quality of life without imposing significant additional financial burdens.

Community-based programmes demonstrated cost-related benefits. Chirico *et al*^23^ analysed ‘CompassionNet’, a multidisciplinary community-based programme including paediatric nurse practitioners, child life specialists and other providers offering support. They reported substantial savings from reduced hospital inpatient use attributed to children enrolled on the programme being more likely to die at home: death in the hospital ($22 004 PPPM) incurred substantially higher costs than death at home ($13 323 PPPM). It was noted that children with malignancies were more likely to die at home than hospital (56%) compared with other diagnoses (35%).

Lyseki *et al*^31^ compared specialist paediatric palliative care with generalist paediatric palliative care in Canada and found the former associated with lower total healthcare costs ($5246 PPPM vs $6690 PPPM) and fewer acute and intensive care unit (ICU) days, indicating more efficient resource utilisation.

Home-based hospice care presented another avenue for cost savings. Chong *et al*^29^ evaluated a home-based hospice service and found reduced hospital admissions and lengths of stay, suggesting cost savings of $12 717 PPPM in the last year of life. This remained economically beneficial if costs of the 24/7 helpline and human resources were included. Additionally, Gans *et al*^15^ compared a hospice-like service offering comprehensive palliative support with traditional hospice care. The specialised service incurred lower monthly costs ($16 498) compared with usual care ($20 962), primarily due to decreased hospital use and increased reliance on community services, resulting in savings of $4131 PPPM.

Noyes *et al*^30^ employed a multimethod study to estimate service costs and end-of-life care needs of children with life-limiting conditions. The authors identified a broad range of specialist end-of-life services, including paediatric palliative care nurses, medical equipment technicians, clinical psychologists, administrative support and 24/7 telephone nurse consultations. The average annual cost per child (in GBP£) varied significantly, ranging from £2437 to £22 771, depending on the prevalence estimate years (2007 vs 2012/2013). This study also estimated minimum costs of providing 1 week of end-of-life care as £14 000 per child (2010/2011 prices). The study highlighted that end-of-life care and palliative care needs for children can vary widely, reflected in the costing estimates.

## Condition types: CCCs

Nine studies focused on patients described as having CCCs.^16^,^24^ The reported costs ranged from $4866 PPPM for hospice, inpatient and outpatient services,^17^ to $16 350 PPPM for the top 10% most costly children hospital inpatients.^19^ Most studies that included patients with CCC reported costs at the higher end of this range (table 7).

Ananth *et al*^20^ conducted a retrospective cohort analysis using the Paediatric Health Information System to explore hospital resource use and major medical interventions for children with life-threatening CCCs. They found that the overall cost for patients was $16 157 PPPM. When broken down by specific conditions, haematologic/immunologic conditions incurred the highest costs at $61 200 PPPM, and neuromuscular conditions incurred the lowest at a mean cost $12 353 PPPM. They also observed that children with haematological conditions had the highest median number of hospital days in their last year of life (99 days), compared with those with neuromuscular conditions, who had a median of 24 days.

In the same study, children with renal conditions spent a median of 19 days in the ICU, whereas those with malignancies spent a median of 6 days. In terminal admissions, 76% of children were mechanically ventilated, with the majority (87.1%) of these cases involving cardiovascular conditions. The lowest ventilation rates were observed in malignancies. Furthermore, children with three or more CCCs experienced at least four hospitalisations in their last year of life. It is important to note that costs related to outpatient care, community care, hospice or children’s hospitals were not included, as these services were not captured in the administrative data used.

Chirico *et al*^23^ compared the costs of palliative care between children with malignancy conditions and those with other CCCs. Their findings indicated that the cost of palliative care for children with malignancies was significantly higher at $21 401 PPPM compared with $13 699 PPPM for all other conditions. This highlights the substantial financial burden associated with malignancy conditions within this population.

Widger *et al*^21^ conducted a population-based retrospective cohort study using administrative databases to evaluate healthcare costs based on age group and cause of death (perinatal/congenital, chronic, external) over a 3-year period from 2010 to 2013. The study revealed that a higher proportion of perinatal/congenital deaths (62%) occurred in ICU compared with deaths from chronic conditions (40.8%). Most of these deaths took place in acute care settings, with individuals spending an average of 30 days in the hospital during their last year of life for perinatal/congenital conditions and 90 days for those with chronic conditions. Mean expenditures were highest for perinatal/congenital conditions in the last year of life. Specifically, costs nearly doubled between 12 and 5 months before death and increased by 2.5 times in the final 4 months of life, and mean costs tripled in the last 4 months of life for patients with chronic conditions. These findings highlight the substantial escalation in healthcare costs as death approaches, particularly for perinatal/congenital and chronic conditions.

## Costs associated with cancer

Four studies specifically investigated the costs of care for children with cancer, revealing variations in costs based on cancer type (table 7).^25^,^28^ As a general trend, the costs of care for children with cancer in the last year of life were higher than the costs associated with other CCCs. Three studies analysed the costs associated with different cancer types, including inpatient hospitalisations, ED visits, outpatient chemotherapy visits and same day surgery. All consistently found that patients with leukaemia incurred the highest costs.^25^,^27^ Specifically, the costs for leukaemia ranged from a mean of $25 191 for adolescents (aged 15–19.9 years),^25^ to a median of $54 505.^27^

de Oliveira *et al*^25^ identified costs of care for children and adolescents with cancer 90 days prediagnosis, and the first year following diagnosis. Patients were followed a year after diagnosis, or from diagnosis to death, and matched with non-cancer patient controls. Costs were higher for children (aged 91 days–14 years) who incurred costs of $19 105 PPPM compared with $16 747 PPPM incurred by adolescents (aged 15–19.9 years). Hospitalisation accounted for 82% of all costs, followed by physician services, chemotherapy and then radiation. de Oliveira *et al*^26^ also reported the costs broken down by cancer types, revealing leukaemia to be the costliest at $48 036 PPPM, and the category ‘all other cancers’ the least costly ($27 813 PPPM). The authors found that the difference in costs between cancer types was driven by inpatient hospital stays, which accounted for 76% of all costs, followed by physician services and chemotherapy.

Nathan *et al*^27^ assessed healthcare use and costs for adolescents aged 15–19 years with cancer, comparing those receiving care in adult versus paediatric institutions. Costed resources included ED visits, hospitalisations, outpatient chemotherapy and homecare across four distinct phases: prediagnosis (60 days before diagnosis), initial (360 days after diagnosis), terminal (360 days before death) and continuing (time between initial and terminal/end of observation). While overall resource use was higher in paediatric centres, hospital use during the terminal phase was similar between paediatric and adult centres. The highest costs were incurred by children with leukaemia ($54 505 PPPM) and the lowest costs by children with central nervous system cancer ($14 661 PPPM).

McFerran *et al*^28^ estimated the costs of unscheduled care for people with cancer in their last year of life using a retrospective cost consequence prevalence-based approach of secondary data. Costed resources included palliative care support, hospital bed per day and general practioner (GP)/ ambulance involvement per admission. Most patients in the study were adults, with fewer than 10 children (aged 0–19 years) included in the analysis. The authors found that children had the highest number of inpatient days per year (25 days), with a mean cost of $1290 PPPM. Overall, the authors reported that unscheduled care in the last year of life for cancer is significant and costly for acute health services—although with only 5% of costs associated with input from specialist inpatient palliative care.

## Discussion

Despite varying research objectives, methodologies, resource measures, data sources and child populations (age and condition), key novel findings were as follows: specialised paediatric palliative care interventions generally led to cost efficiencies by reducing hospitalisations, length of stay and optimising resource use.^15 19 23 29 31 34^ These patterns highlight the potential for specialised palliative care to enhance both clinical outcomes and efficiency in paediatric healthcare. Specialist paediatric palliative care could also add value to inpatient healthcare that focuses on quality of life and may be less expensive when children are near death, by lowering costs and simultaneously increasing the likelihood of children dying at home.^35^ This may also enhance the quality of life for children and their families.^36^ For example, children receiving palliative care on discharge from paediatric ICU (PICU) were found to be eight times more likely to die in the community compared with those without a palliative care referral.^7^

Total costs in the last year of life were highest for children with cancer compared with other life-limiting conditions, although children with malignancies were more likely to die at home than hospital (56%) than other diagnoses (35%).^23^ Leukaemia was identified as the most expensive cancer from a health service resource perspective in the last year of life.^25^,^27^ A review by Nabukalu *et al*^37^ similarly highlighted that healthcare costs for children with cancer were highest for those diagnosed with leukaemia, both in the first and last year of life.^37^ Hospitalisation expenses accounted for over one-third of the total healthcare costs. This reflects the ‘U’-shaped cost trend observed in children with cancer: the highest costs occur at the beginning and end-of-life, with lower costs during the intermediate phase of illness.^25 37^ However, further research is needed to explore specialist palliative care in improving symptom management and quality of life for children with cancer.^38^

The intensity of care in the final months of life significantly increased costs, especially among those with malignancies and multiple CCCs.^20 21^ Higher costs are incurred in hospital settings, where most children with a life-limiting condition continue to die.^39 40^ Neurological conditions account for around 60% of all PICU admissions.^7^

Infants are more likely to die in hospital than home compared with older children.^39^ Younger children typically require more intensive and costly care compared with older children and adolescents.^16 22 25 33^ Radiotherapy and other treatments for younger children can be resource intensive and costly due to extra time, personnel and anaesthesia.^21^

Several studies suggested a future research focus on recording and integrating quality of care, quality of life and parent-reported outcomes to help understand the effects of increased surgeries and invasive treatments (eg, gastrostomy and tracheostomy) on the child and their family at the end-of-life.^20 21 24 29 31^ Costs to families (time and money) are substantial even where there is universal healthcare coverage. Therefore, the significant financial burden on the family warrants further investigation.^25 26 41^ Furthermore, post-death support is a crucial element of palliative care and can include resources and support from specialist staff for families to enable the parent to spend time with their child after death.^42 43^ It is recommended that this should be recognised in future research investigating costs in the last year of life.

### Limitations

We chose to focus only on high-income countries that were broadly comparable to the UK healthcare system, although with different funding models. A range of resources were identified, with costs derived from different sources using varied currencies and calculations. From a cost perspective, some studies included only healthcare expenses, while others also accounted for costs related to non-profit hospice care and social services. Further limitations included relying on commercial insurance data and Medicaid claims data, which only covered a subset of the population.^16^,^1822^ This lack of standardisation meant that direct comparisons and generalisations across studies could not be made, and a robust estimate of costs PPPM for the last year of life could not be calculated.

This is perhaps unsurprising considering that even within one country, variations/heterogeneity of studies can make reviewing the evidence on the cost of palliative care a challenge.^44^ To identify the existing evidence on costs in the last year of life, the research question was deliberately broad, nonetheless, only 20 studies were included, which suggests a paucity of research in this area.

## Conclusion

This comprehensive review provides novel findings and insights. While some trends were identified across studies, which will be helpful when commissioning services, a robust estimate of costs PPPM could not be achieved. This review demonstrates the need for future studies to explore and report on the resources, services and costs to support future planning and resource allocation.

## Supplementary material

10.1136/bmjpo-2025-003526 online supplemental file 1

10.1136/bmjpo-2025-003526 online supplemental file 2

10.1136/bmjpo-2025-003526 online supplemental file 3
