# Supplementary material for: Economic costs of health and social care for a child with a life-limiting condition in their last year of life: a systematic review
Source: BMJ Paediatr Open. 2025 Jul 16;9(1):e003526. doi: 10.1136/bmjpo-2025-003526 (PMC12273097; doi:10.1136/bmjpo-2025-003526)
Supplement: online supplemental file 1 [file bmjpo-9-1-s001.docx]

Supplementary file. 1

**The economic cost of health and social care for a child with a life-limiting condition in their last year of life: A systematic review**

**Background:** There are an estimated 21 million children who require palliative care globally, with over 97% in low-middle income countries^[[1]](#footnote-1)^. Children’s palliative care aims to enhance the quality of life of children with life-threatening conditions and their families. Described as “*an active and total approach to care, from the point of diagnosis or recognition throughout the child’s life, death and beyond*.”^[[2]](#footnote-2)^ This can involve managing distressing symptoms as well as short break provision. Unlike adult palliative care, care ideally starts at diagnosis and can be for a few days to years^[[3]](#footnote-3)^. The population and prevalence of children needing palliative care is changing. Advances in medicine and technology have reduced mortality, and there are increasing health and care needs. Moreover, whilst there are archetypes of life-limiting conditions^[[4]](#footnote-4)^ as well as phases/trajectories there is no ‘typical pathway’^[[5]](#footnote-5)^ for paediatric palliative care needs. To plan for effective children’s palliative care services and estimate future care costs, current costs need to be identified.

**Aim:** This systematic review aims to review and descriptively synthesise all literature reporting on the costs associated with any health and/or social care services provided to children with a life-limiting condition, in their last year of life.

**Design:** Search terms include Palliative care OR Palliative Medicine OR Hospice* AND Cost* OR Economic* OR Cost-Benefit Analysis OR life-limiting OR life threatening AND Health Care Costs OR Budget* OR Health expenditures

Databases searched will include MEDLINE, EMBASE, Cochrane Library, and CINAHL. The specialist database of children’s palliative care studies will also be searched, including the Together for Short Lives website for children and young people’s palliative care (www.togetherforshortlives.org.uk) and paediatric palliative care library <https://pedpalascnetlibrary.omeka.net/>.

**Eligibility criteria:** Inclusion criteria: palliative end of life care services for children (age <19) in healthcare systems comparable to the UK. Primary outcomes(s): reporting on economic costs associated with paediatric palliative and end of life care. Language: English or Welsh. Dates: January 2004 to present. Published and preprint articles. Exclusion criteria: palliative or end of life care, models and/ or services for adults only (age >19). Countries with healthcare systems dissimilar to UK models of care. Studies not including economic evidence of paediatric palliative care. Languages other than English or Welsh. Protocols, editorials, letters, commentaries.

**Data extraction:** Data extraction will be based on the outlined eligibility criteria. We will extract details/characteristics on study country, study design, type of service, participants (age and archetype), costs, outcomes, and study settings.

**Quality appraisal:** Members of the research team will independently screen titles, abstracts, and full texts for relevance. Disagreements will be resolved through discussion. The Drummond checklist will be used to critically appraise studies^[[6]](#footnote-6)^.

**Synthesis:** A narrative synthesis of the findings will be presented including costs of services.

1. Worldwide Palliative Care Alliance. Global atlas of palliative care. London: Worldwide Palliative Care Alliance; 2020. [↑](#footnote-ref-1)
2. Together for Short Lives. A guide to children’s palliative care. Together for Short Lives: Bristol; 2018 [↑](#footnote-ref-2)
3. Fraser LK, Bluebond-Langner M and Ling J. Advances and challenges in European paediatric palliative care. Med Sci (Basel) 2020;8. [↑](#footnote-ref-3)
4. Association for Children’s Palliative Care (ACT)/Royal College of Paediatrics and Child Health (RCPCH) A guide to the development of children’s palliative care services: report of the joint working party. Bristol, 1997 [↑](#footnote-ref-4)
5. Wood F, Simpson S, Barnes E, Hain R. Disease trajectories and ACT/RCPCH categories in paediatric palliative care. Palliat Med. 2010 Dec;24(8):796-806 [↑](#footnote-ref-5)
6. Drummond M, Sculpher M, Torrance G, O'Brien B, Stoddart G. Methods for the economic evaluation of health care programs. Oxford: Oxford University Press; 2005. [↑](#footnote-ref-6)
