# Supplementary material for: Economic costs of health and social care for a child with a life-limiting condition in their last year of life: a systematic review
Source: BMJ Paediatr Open. 2025 Jul 16;9(1):e003526. doi: 10.1136/bmjpo-2025-003526 (PMC12273097; doi:10.1136/bmjpo-2025-003526)
Supplement: online supplemental file 2 [file bmjpo-9-1-s002.docx]

Supplementary file 2.

**Search strategy Medline (via OVID) database**

**Date:** 08.10.2024

1. Palliative Care/

2. Terminally Ill/

3. Terminal Care/

4. Hospice Care/

5. life limiting condition.mp.

6. life limiting illness.mp.

7. Pediatrics/

8. paediatric.mp.

9. Child/

10. Adolescent/

11. Infant, Newborn/

12. "Health Care Economics and Organizations"/

13. Models, Economic/ or Cost-Benefit Analysis/ or Health Care Costs/ or Budgets/

14. "Delivery of Health Care"/

15. "Cost Savings"/

16. life threatening condition.mp.

17. exp "costs and cost analysis"/ or budgets/

18. 1 or 2 or 3 or 4 or 5 or 6 or 16

19. 7 or 8 or 9 or 10 or 11

20. 12 or 13 or 14 or 15 or 16 or 17

21.18 and 19 and 20

22. limit 21 to yr="2004 - 2025"

**Table S1.** Sources of Country-Level Consumer Price Index (CPI) Data

| **Country** | **Data Source** |
| --- | --- |
| Canada | Bank of Canada. Inflation Calculator - Bank of Canada. Retrieved January 28, 2025, from <https://www.bankofcanada.ca/rates/related/inflation-calculator/> |
| United States of America | US Bureau of Labor Statistics. CPI Inflation Calculator. Retrieved January 28, 2025, from https://data.bls.gov/cgi-bin/cpicalc.pl?cost1=1.00&year1=201301&year2=202401 |
| United Kingdom | Bank of England. Inflation calculator - Bank of England. Retrieved January 28, 2025, from <https://www.bankofengland.co.uk/monetary-policy/inflation/inflation-calculator> |
| Singapore | World Data. Inflation rates in Singapore. Retrieved January 28, 2025, from https://www.worlddata.info/asia/singapore/inflation-rates.php |

**Table S2.** Reported year for costing data

| **Study** | **Costing base year** |
| --- | --- |
| Ananth et al (2015) | 2012 |
| Chirico et al (2019) | 2015 |
| Chong et al (2018) | 2015 |
| Cozad et al (2022) | 2013 |
| de Oliveira et al (2017a) | 2012 |
| de Oliveira et al (2017b) | 2012 |
| Gans et al (2016) | 2013 |
| Knapp et al (2009) | 2006 |
| Lemoine et al (2022) | 2009 |
| Lindley et al (2013) | 2007 |
| Lindley et al (2019) | 2014 |
| Lindley et al (2022) | 2013 |
| Lysecki et al (2022) | 2014 |
| Nathan et al (2019) | 2012 |
| Noyes et al (2013) | 2007 |
| McFerran et al (2023) | 2015 |
| Smith et al (2015) | 2010 |
| Svynarenko et al (2022) | 2013 |
| Svynarenko et al (2024) | 2013 |
| Widger et al (2017) | 2013 |

**Table S3.** Currency Exchange Rates Relative to the US Dollar (USD)

| **Currency** | **Exchange Rate to United States Dollar (USD)** |
| --- | --- |
| British Pound (GBP) | 1 GBP = 1.27 USD |
| Canadian Dollar (CAD) | 1 CAD = 0.71 USD |
| Singapore Dollar (SGD) | 1 SGD = 0.75 USD |
