# Supplementary material for: Economic costs of health and social care for a child with a life-limiting condition in their last year of life: a systematic review
Source: BMJ Paediatr Open. 2025 Jul 16;9(1):e003526. doi: 10.1136/bmjpo-2025-003526 (PMC12273097; doi:10.1136/bmjpo-2025-003526)
Supplement: online supplemental file 3 [file bmjpo-9-1-s003.docx]

**Table S4.** Quality assessment of included studies using the Drummond Checklist

| **Study** | **1.**  **Research question well defined?** | **2. Comprehensive description of alternatives?** | **3.**  **Effectiveness of program established?** | **4.**  **Important & relevant costs & consequences for each alternative identified?** | **5.**  **Costs & consequences measured accurately & appropriately?** | **6.**  **Costs & consequences valued credibly?** | **7.**  **Costs & consequences adjusted for differential timing?** | **8.**  **Incremental analysis of costs & consequences performed?** | **9.**  **Allowance made for uncertainty in estimates?** | **10.**  **Presentation on & discussion of study results include all issues of concern to users?** |
| --- | --- | --- | --- | --- | --- | --- | --- | --- | --- | --- |
| *Ananth et al (2015)* | Yes | Partially yes | NA | Yes | Yes | Yes | Yes | No | Partially yes | Yes |
| *Chirico et al (2019)* | Yes | Partially yes | Partially yes | Partially yes | Yes | Partially yes | NA | Partially yes | Partially yes | Yes |
| *Chong et al (2018)* | Yes | Yes | Yes | Yes | Yes | Yes | NA | Yes | Partially yes | Yes |
| *Cozad et al (2022)* | Yes | Yes | Partially yes | Yes | Yes | Yes | No | Yes | Partially yes | Yes |
| *de Oliveira et al (2017a)* | Yes | Yes | Yes | Yes | Yes | Yes | No | Yes | Yes | Yes |
| *de Oliveira et al (2017b)* | Yes | Yes | NA | Yes | Yes | Yes | Yes | Yes | Yes | Yes |
| *Gans et al (2016)* | Yes | Partially yes. | Partially yes | Yes | No | Yes | No | NA | Partially yes | Yes |
| *Knapp et al (2009)* | Yes | No | NA | NA | Yes | Yes | NA | No | Partially yes | Yes |
| *Lemoine et al (2022)* | Partially yes | Yes | Partially yes | Partially yes | Yes | Yes | No | No | Partially yes | Yes |
| *Lindley et al (2013)* | Partially yes | No | NA | Yes | Yes | Yes | No | No | No | Yes |
| *Lindley et al (2019)* | Yes | No | NA | No | Yes | Yes | No | No | No | Yes |
| *Lindley et al (2022)* | Yes | Yes | Partially yes | Yes | Yes | Yes | Yes | Yes | Yes | Yes |
| *Lysecki et al (2022)* | Yes | Yes | Partially yes | Partially yes | Yes | Yes | NA | Yes | Partially yes | Yes |
| *Nathan et al (2019)* | Yes | Partially yes | Partially yes | Yes | Partially yes | Yes | No | Partially yes | Partially yes | Yes |
| *Noyes et al (2013)* | Yes | Partially yes | Partially yes | Yes | Yes | Yes | No | Partially yes | Partially yes | Yes |
| *McFerran et al (2023)* | Yes | Partially yes | Partially yes | Yes | Yes | Yes | Yes | Yes | Partially yes | Yes |
| *Smith et al (2015)* | Yes | No | Partially yes | Yes | Yes | Yes | No | No | Partially yes | Yes |
| *Svynarenko et al (2024)* | Yes | Yes | Partially yes | Yes | Yes | Partially yes | Partially yes | Yes | Partially yes | Yes |
| *Svynarenko et al (2022)* | Yes | Yes | Partially yes | NA | Yes | No | No | Yes | Yes | Yes |
| *Widger et al (2017)* | Yes | Partially yes | NA | Yes | Yes | Yes | Partially yes | Yes | No | Yes |
