# Supplementary material for: Economic costs of health and social care for a child with a life-limiting condition in their last year of life: a systematic review
Source: BMJ Paediatr Open. 2025 Jul 16;9(1):e003526. doi: 10.1136/bmjpo-2025-003526 (PMC12273097; doi:10.1136/bmjpo-2025-003526)
Supplement: Supplementary file 4 [file bmjpo-2025-003526.reviewer_comments.pdf]

PEER REVIEW HISTORY

BMJ Paediatrics Open publishes all reviews undertaken for accepted manuscripts. Reviewers are asked to complete a checklist review form and are provided with free text boxes to elaborate on their assessment. These free text comments are reproduced below.

ARTICLE DETAILS

**TITLE (PROVISIONAL)**	The economic costs of health and social care for a child with a life-limiting condition in their last year of life: A systematic review.
**AUTHORS**	Prendergast, Louise; Crane, Ellie; Bray, Nathan; Noyes, Jane

VERSION 1 - REVIEW

**REVIEWER NAME**	***Ammar Abbas***
**REVIEWER AFFILIATION**	University of Baghdad Bab Al-Moadham Campus College of Nursing, pediatric nursing
**REVIEWER CONFLICT OF INTEREST**	N/A
**AI DISCLOSURE**	No
**DATE REVIEW RETURNED**	03-Jun-2025

**GENERAL COMMENTS**	To the Respected Writers,<br><br>As a reviewer, I would like to say how much I value your research paper. Your attention to the financial strains of caring for children in their final year of life who have life-limiting illnesses is a vital addition to the body of literature. It is critical to comprehend these expenses and how they affect the lives of children and their families. Your work offers priceless insights that can help create healthcare and social care policies that are more equitable and successful.<br>All things considered, I think this study has substantial practical significance in addition to being of excellent scientific quality. It offers a strong starting point for additional study and conversations about how to lessen the financial strain on families dealing with these difficult situations and enhance

**REVIEWER NAME**	***Angelica Chantre***
**REVIEWER AFFILIATION**	None disclosed
**REVIEWER CONFLICT OF INTEREST**	N/A
**AI DISCLOSURE**	No
**DATE REVIEW RETURNED**	08-Jun-2025

**GENERAL COMMENTS**	This is a very interesting topic that should be carefully evaluated, since public health policies in different countries are based on evidence from cost-effectiveness studies. As mentioned in the discussion, it is worth evaluating the quality of care, quality of life, and other factors related to the diseases included in the study. I would highlight the initial paragraph of the discussion, in which it is mentioned that, in general terms, palliative care reduces health costs by reducing hospitalizations and improving the quality of life of patients and their families who receive palliative care at home. I

	would recommend, if possible, mentioning the concept of Complex Chronic Conditions and which diseases are included in the methodology description. For future studies, the possibility of expanding this type of research to evaluate the quality and costs of palliative care services in developing countries is raised.<br>Thank you for your work and contributions
